# Different but Not Unique: Deciphering the Immunity of the Jamaican Fruit Bat by Studying Its Viriome

**DOI:** 10.3390/v14020238

**Published:** 2022-01-25

**Authors:** Quinnlan David, Tony Schountz, Martin Schwemmle, Kevin Ciminski

**Affiliations:** 1 Institute of Virology, University Medical Center Freiburg, 79104 Freiburg, Germany; quinnlan.david@uniklinik-freiburg.de (Q.D.); martin.schwemmle@uniklinik-freiburg.de (M.S.); 2 Faculty of Medicine, University of Freiburg, 79104 Freiburg, Germany; 3 Spemann Graduate School of Biology and Medicine (SGBM), University of Freiburg, 79104 Freiburg, Germany; 4 Faculty of Biology, University of Freiburg, 79104 Freiburg, Germany; 5 Department of Microbiology, Immunology and Pathology, College of Veterinary Medicine and Biomedical Sciences, Colorado State University, Fort Collins, CO 80523, USA; Tony.Schountz@colostate.edu

**Keywords:** bat viruses, *Artibeus jamaicensis*, Tacaribe virus (TCRV), rabies virus (RABV), bat influenza A virus (IAV) H18N11, bat infection studies, bat immunity

## Abstract

A specialized and fine-tuned immune response of bats upon infection with viruses is believed to provide the basis for a “friendly” coexistence with these pathogens, which are often lethal for humans and other mammals. First insights into the immunity of bats suggest that bats have evolved to possess their own strategies to cope with viral infections. Yet, the molecular details for this innocuous coexistence remain poorly described and bat infection models are the key to unveiling these secrets. In Jamaican fruit bats *(Artibeus jamaicensis)*, a New World bat species, infection experiments with its (putative) natural viral pathogens Tacaribe virus (TCRV), rabies virus (RABV), and the bat influenza A virus (IAV) H18N11, have contributed to an accurate, though still incomplete, representation of the bat-imposed immunity. Surprisingly, though many aspects of their innate and adaptive immune responses differ from that of the human immune response, such as a contraction of the IFN locus and reduction in the number of immunoglobulin subclasses, variations could also be observed between Jamaican fruit bats and other bat species.

## 1. Introduction

The Jamaican fruit-eating bat, *Artibeus jamaicensis*, is a frugivorous bat species that is native to Central America. Its main habitats are the rainforests extending from Southern Mexico throughout the Caribbean and northern regions of South America, yet they inhabit a wide range of elevations from subalpine to sea level [1,2]. Jamaican fruit bats can reach a weight of up to 60 g, with a wingspan of just 96–150 mm; they can live up to ten years and birth twice a year, generally giving birth to only a single pup [1,2]. Like all other bats, they have an extraordinarily high metabolism for their size that requires a constant calorie intake [3]. Characteristic of these bats is their nose-leaf, a facial organ that is used for echolocation, including communication, foraging, and detection of predators. As a fruit-eating bat, their diet consists mainly of *Ficus* spp. figs, although they will occasionally resort to eating flowers and insects if fruit is unavailable [2,4]. As a result, these bats are key contributors to seed dispersal, pollination and pest control in their environment. Additionally, they represent an important food source for a variety of predators, including owls, snakes, and even other bats [1].

A number of studies since the 1950s found that Jamaican fruit bats have an important role in viral ecology as well. They were found to be infected with multiple viruses in nature, including dengue virus (DENV) [5,6,7], West Nile virus (WNV) [8], Zika virus (ZIKV) [8], alphacoronaviruses (α-CoV) [9], chikungunya virus (CHIKV) [10], Tacaribe virus (TCRV) [11,12], and rabies virus (RABV) [13,14,15,16], many of which are pathogenic to humans. Although infections with DENV, WNV, and ZIKV are due to epizootic events, it could be shown that Jamaican fruit bats may represent a reservoir for RABV [15]. TCRV, which has been isolated from bats of two *Artibeus* species, did not transmit to other bats under experimental conditions [17]. In contrast, genomic sequences of bat influenza A virus (bat IAV) subtype H18N11 were detected in multiple *Artibeus* species and the virus was, moreover, found to serially transmit between experimentally infected Jamaican fruit bats, consistent with its role as a putative reservoir species. Studying the relationship of these viruses and their (putative) natural host, the Jamaican fruit bat, has shed some light into the mechanisms of how these bats can tolerate viral infections and avoid severe symptoms of disease in contrast to dead end hosts such as humans.

## 2. Infection Studies

### 2.1. Tacaribe Virus

TCRV is an arenavirus that was first isolated in the 1950s, reportedly from Jamaican fruit bats and great fruit-eating bats (*Artibeus lituratus)* in Port-of-Spain, Trinidad [12]. Subsequent research using molecular tools has determined that Jamaican fruit bats are not found in Trinidad, but instead it is likely that these were misidentified flat-faced fruit bats *(Artibeus planirostris)* [18]. In total, TCRV was isolated from six great fruit-eating bats and five flat-faced fruit bats in the 1950s, suggesting a widespread distribution of these viruses among *Artibeus* spp. Despite efforts to preserve all eleven isolates, only one of these viruses remains: TRVL-11573, which was isolated from a great fruit-eating bat [19]. Antibodies to TCRV have also been found in other fruit-eating bats, such as the Seba’s short-tailed bat *(Carollia perspicillata)* and the yellow-shouldered fruit bat *(Sturnira lilium)* [11]. Furthermore, as TCRV has otherwise only been isolated from ticks in northern Florida, USA (where fruit bats are not found) [20], it is suspected to be the only New World arenavirus that has no known rodent reservoir [17].

Experimental infection of Jamaican fruit bats with a low dose of TCRV (10^4^ TCID_50_), either subcutaneously or via an intranasal route, resulted in an asymptomatic, apathogenic infection, and virus clearance. In contrast, a high dose of virus (10^6^ TCID_50_) administered subcutaneously or intranasally resulted in severe neurological disease (e.g., poor responses to mechanical stimuli and incoordination, as well as wing, ear, and head tremors), an inability to fly, and high mortality. Despite a clear pattern of virus progression in high-dose infected bats, TCRV-specific RNA and infectious virus were first identified in the spleen before their detection in various other organs, including the lungs, liver, intestine, and brain, the latter of which appeared to be especially associated with a fatal outcome. Histopathological analysis revealed that the bats suffered from pneumonia together with tissue damage present in the liver and spleen, as well as (in most cases) lesions in the brain [17]. Intriguingly, horizontal transmission experiments demonstrated that TCRV was unable to spread between inoculated donor bats and naïve contact animals, leading to the assumption that Jamaican fruit bats might not be the natural reservoir species of TCRV [17]. Nevertheless, it remains to be determined how TCRV infects *Artibeus* bats in nature and how it achieves widespread circulation among these bats.

Subsequent transcriptome studies of the spleen, liver, and kidneys of infected Jamaican fruit bats revealed organ-specific differences in the induction of the innate and adaptive immune responses [21]. Intriguingly, among the most prominently regulated pathways was the interferon (IFN)-signaling pathway, of which most of its related genes (*Ifit1*, *Ifit3*, *Ifi6*, *Irf9*, *Bax*, *Socs1,* and *Bcl2*) were upregulated in at least two of the three tissues analyzed. Furthermore, transcripts for type II IFN, but not type I IFN, were increased over the uninfected controls. Additionally, transcripts for cytokine (*Il6*, *Il8*, *Il1a*, and *Il1b*) and chemokine genes (*Cxcl1*, *Cxcl2*, *Cxcl3*, *Cxcl5*, and *Cxcl6*) were elevated. Expression signatures of apoptosis, which is generally associated with antiviral activity, was increased in the kidneys, though decreased in the spleen and liver, based on transcript levels for the anti-apoptotic marker BCL-2. Sera collected from both high- and low-dose infected bats were mostly negative for neutralizing antibodies. However, three bats, in which TCRV-specific RNA was not detected, tested positive for neutralizing antibodies. Despite abundant expression of transcripts coding for the immunoglobulin (Ig) isotypes IgM, IgE, IgA, and IgG in the spleen, infected bats showed no induction of genes involved in somatic hypermutation and affinity maturation, which may account for low antibody titers. As expected, genes associated with T and B lymphocyte activity were upregulated, yet no upregulation was observed in transcripts associated with the activation of NK cells [21].

### 2.2. Rabies Virus

RABV is a rhabdovirus that is found globally in many mammalian species, particularly associated with canines and bats [22]. Infections of humans with RABV are nearly always lethal, even with intervention [23]. In 1974, RABV was isolated from the brain of a disorientated Jamaican fruit bat in La Tanta, Grenada [14]. Consistent with its ability to infect a wide range of animals, the virus circulates in many other *Artibeus* species, including the flat-faced fruit bat and great fruit-eating bat. *Artibeus* bat colonies in Grenada are therefore believed to be reservoirs of RABV [13,14,15,16].

RABV is a lethal neurotropic virus, replicating in the brain and salivary glands. Virus-containing saliva enters the body intramuscularly via a bite, scratch, or while grooming, after which it progresses to the central nervous system [24]. In accordance with this, a previous study using a mouse-adapted RABV strain CVS-24, found that intramuscular infection of Jamaican fruit bats with a high-virulence strain (CVS-24-N2c) into the right masseter muscle led to widespread inflammation in the central nervous system, characterized by lymphocyte infiltration, particularly in the brain stem, leptomeningeal, and perivascular regions. By day seven post-infection, bats infected with CVS-24-N2c developed ataxia, leg paresis, and an inability to fly, whereas bats infected with the low-virulence strain CVS-24-B2c showed no symptoms of disease [25]. In contrast to the observations made using the murine-derived CVS-24-N2c virus, intramuscular and subcutaneous infection of the intermediate fruit bat *(Artibeus intermedius)* with different vampire bat-derived RABVs did not cause clinical signs of disease and most of the experimentally infected bats seroconverted with neutralizing antibodies [26]. However, studies investigating the natural route of transmission are currently lacking and, given the special features of the central nervous system and its quiescent immune status [27], immunological studies are hindered [28].

Recent serological examinations that aimed to identify exposure of bats to RABV in Trinidad revealed flat-faced fruit bats, at the time misidentified as Jamaican fruit bats, to be most seropositive of the species tested [15]. Interestingly, it appeared that juvenile bats were more likely to be seropositive than adult bats. Urban areas were also more likely to contain seropositive bats than more rural regions. Moreover, despite the presence of neutralizing antibodies (titers ranging from 10 to 1900), these bats did not display signs of disease [15], indicating that unlike the experimental infection experiments using the murine CVS-24-N2c strain, the infection was not lethal to these wild bats. Studies have shown, notably, that RABV strains of bat origin tend to be less pathogenic in mice than strains from other reservoirs, indicating an adaptation of the virus to its host [29]. Based on cases reported of transmission between bats and livestock close to Jamaican fruit bat colonies, RABV circulating in these bats is considered a public health concern in certain regions of Grenada, however, its pathogenicity in comparison to RABV from other species is yet to be determined.

### 2.3. Bat Influenza A Virus H18N11

In 2010, a genome sequence belonging to a previously unknown bat IAV of the H18N11 subtype was isolated from flat-faced fruit bats in the Peruvian Amazonia. In the following years, H18N11 genome sequences were also found in dark fruit-eating bats *(Artibeus obscurus)* from Bolivia and great fruit-eating bats from Brazil, indicating a widespread circulation of the H18N11 virus among *Artibeus* spp. [30].

Following intranasal inoculation of Jamaican fruit bats, a likely reservoir of bat IAV [30], with 5 × 10^5^ TCID_50_ of H18N11, some bats displayed mild clinical signs of disease, such as nasal and ocular discharge. However, unlike conventional IAVs (subtypes H1-16 and N1-9), which predominantly replicate in the epithelium of the respiratory organs, the bat IAV H18N11 targeted the squamous epithelium of the palatine tonsils, the lamina propria of the small intestine, and the follicle-associated epithelium of the jejunal Peyer’s patches. Histological staining of the infected intestinal tissue revealed viral RNA in both enterocytes and antigen-presenting cells (macrophages or dendritic cells). In line with the observed intestinal replication, infected bats shed virus-containing feces, resulting in a most likely fecal–oral horizontal transmission of the virus to naïve contact bats [31]. Previous reports of naturally infected flat-faced fruit bats and great fruit-eating bats similarly reported intestinal replication and shedding of H18N11 in rectal specimens [30,32]. The unconventional ability of the bat IAV to infect and replicate in lymphoid tissue, which is occupied by a high number of leukocytes, is due to its hemagglutinin surface glycoprotein H18, that uses the major histocompatibility complex class II (MHC-II) proteins for cell entry [33]. In the context of the Jamaican fruit bat infection, the neuraminidase protein N11, the second surface glycoprotein of the bat IAV, appeared to be crucial for viral transmission. Subsequent in vitro experiments revealed that N11 negatively regulated MHC-II expression [31], presumably to facilitate viral egress. Considering the cellular tropism of H18N11 in antigen-presenting cells, the immunological consequences in terms of CD4^+^ T cells need to be addressed.

Serological testing of bats captured in the Peruvian rainforest region showed, firstly, that the proportion of seropositive bats was highest among *Artibeus* spp. and, secondly, the presence of IgGs directed to the H18 HA and N11 NA surface glycoproteins, with titers of 1000 and, in some bats, even 16,000 [30]. Upon experimental infection of Jamaican fruit bats, index and contact bats similarly seroconverted with antibodies to the viral nucleoprotein with titers of 300–1000 (antibodies to HA and NA were not tested in this study) [31].

## 3. Discussion

Jamaican fruit bats are a widely distributed New World bat species that share overlapping habitats with multiple other species and, increasingly, humans. Consequently, as mentioned above, Jamaican fruit bats have been found to be infected with a multitude of different viruses in nature [5,6,8,9,11,13,15,16,30]. Moreover, it has been used as an experimental infection model for several of these viruses, including those for which Jamaican fruit bats constitute a putative natural reservoir, such as TCRV [17,21], RABV [25], and bat IAV H18N11 [31]. These infection experiments revealed specific aspects of the virus–host interplay and the imposed immunity. For instance, low dose TCRV infections, but not high dose infections, allowed viral replication without obvious pathology [17]. Although natural RABV infections caused no obvious pathology, experimental infections with a mouse-derived RABV strain led to severe pathology [14,15,16]. Finally, experimental infection of bats with H18N11 resulted in predominantly intestinal replication, no to very mild signs of disease and efficient fecal–oral transmission [31]. Aside from these studies, Jamaican fruit bats were also used for experimental infections with ZIKV [34], DENV [35,36], and Middle East respiratory syndrome coronavirus (MERS-CoV) [37]. Infection with these viruses neither led to sustained viral replication (with the exception of MERS-CoV) nor caused signs of disease. Nevertheless, the results of these studies, especially regarding the immunological aspects, should be interpreted with caution, as the immune responses observed in these experiments might not be representative for an actual host adaptation to a certain virus. Rather than being an example of virus–host co-evolution, these epizootic infection experiments are prone to generate an unbalanced immune response as seen for humans after infections with zoonotic pathogens [38,39,40,41,42,43].

**Figure 1:**
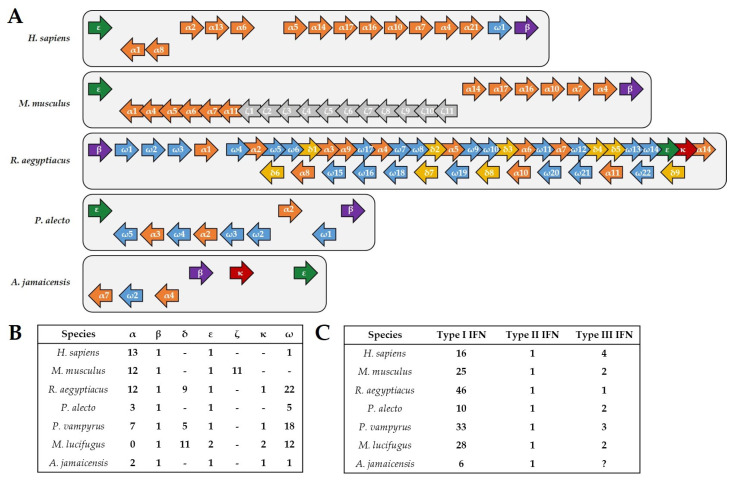
IFN genes in humans, mice and bats. (**A**) Schematic representation of the type I IFN gene loci in humans, mice, *R. aegyptiacus*, *P. alecto*, and *A. jamaicensis*. Although *R. aegyptiacus* exhibits an expansion of the type I IFN genes, both *P. alecto* and *A. jamaicensis* demonstrate a contraction compared to humans and mice. Genes encoding IFN-α are shown in orange, IFN-β in purple, IFN-δ in yellow, IFN-ε in green, IFN-ζ in grey, IFN-κ in red, and IFN-ω in blue. (**B**) Number of the different type I IFN members found in humans, mice, *R. aegyptiacus*, *P. alecto*, *P. vampyrus*, *M. lucifugus*, and *A. jamaicensis*. (**C**) Comparison of the number of genes coding for functional type I, type II, and type III IFN genes for each of the indicated species. Notably, genes encoding IFN-λ have not yet been identified in *A. jamaicensis*.

Although preliminary, the current data available allow for drawing first conclusions about the immunity of Jamaican fruit bats and its differences to other bat species, as well as to humans. Transcriptome studies revealed that the Jamaican fruit bat encodes 466 immune-related genes, corresponding to 2% of the total transcriptome [44]. Similarly, the well-studied Old World black flying fox *(Pteropus alecto)* and Egyptian fruit bat *(Rousettus aegyptiacus)* harbor 500 and 407 immune-related genes corresponding to 3.5% and 2.75%, respectively, of the whole transcriptome [45,46]. In comparison, the human genome has 1562 immune-related genes, comprising 7% of the total transcriptome [47]. One striking difference in immunity that has been described for the black flying fox is the contraction of the type I IFN locus [48]. Only ten intact type I IFN genes could be identified for these bats, in comparison to the 16 known for humans and 25 known for mice (Figure 1 A,B) [45,49]. Jamaican fruit bats appear to follow the tendency observed for the black flying fox, since as many type I IFN pseudogenes (six) as coding genes were identified in its genome, suggesting an evolutionary pressure on the type I IFN loci. However, this gene contraction is dramatically different to the described expansion of the type I IFN genes determined for the New World little brown bat *(Myotis lucifugus)*, 28 genes, as well as the Old World large flying fox (*Pteropus vampyrus)*, 33 genes, and the Egyptian fruit bat, 46 genes [49,50]. Akin to other mammals, there is only one type II IFN (IFN-γ), which has no evolutionary relationship to type I IFN [51], in Jamaican fruit bats, black flying foxes, large flying foxes, Egyptian fruit bats and little brown bats. Although both components of the type III IFN receptor heterodimer, IFNLR1 and IL10R2, were found to be expressed in the Jamaican fruit bat, a functional type III IFN (IFN-λ) gene has not yet been identified in both the NCBI genome database and transcriptomic data (Supplementary Figure S1). However, it cannot be excluded that potential errors in the sequencing data are contributing to the apparent lack of type III interferon in Jamaican fruit bats. If it holds true that type III IFN is subject to negative selection, it would be in contrast to black flying foxes, large flying foxes, little brown bats, and Egyptian fruit bats, for which at least one functional gene encoding IFN-λ has been identified (Figure 1C) [52,53]. Importantly, IL10R2 also forms the heterodimeric receptor for IL-10 and IL-22 [54], both of which are expressed in Jamaican fruit bats. However, the presence of the IFNLR1 component, which is specific to type III IFN signaling [55], is yet to be explained, unless IFN-λ is identified in future studies. Notably, IFN and cytokine receptor components, such as IL10R2 [54], tend to be part of multiple heterodimer receptor complexes which bind different ligands [56]. Therefore, whether a lack of IFN-λ in Jamaican fruit bats hints towards a novel ligand for the receptor component IFNLR1 is a conceivable hypothesis.

A number of previous reports proclaimed the “always-on IFN hypothesis” as one part of an explanation for the special ability of bats to cope with often lethal and human-pathogenic viruses [48,57,58]. For the black flying fox, it was shown that despite constitutive expression of its three IFN-αs, only IFN-β (and not additional IFN-α) levels increased after infection [48]. Because of the constitutive expression of IFNs, the downstream-activated interferon-stimulated genes (ISG) were found to be similarly constitutively active in untreated immortalized black flying fox tissue cultures [48]. Jamaican fruit bats appear to have a constitutive expression of IFN-α and IFN-β as well; however, upon infection with TCRV, only IFN-γ and not type I IFN transcripts were elevated [21]. Moreover, infection led to an upregulation of IFN signaling pathway-related genes and to the induction of *Isg15* and *Irf7* [21]. Interestingly, in the same study, the gene coding for the MHC-II component HLA-DRA was negatively regulated in the kidneys [21]. Thus, the implications for infections with H18N11 that uses MHC-II for cell entry are yet to be evaluated. In contrast, Egyptian fruit bats lack a constitutive IFN expression and immortalized cells of this species respond to viral infection with the induction of IFN-ω and, even more pronounced, IFN-β [49].

A previous study revealed that the IFN response is dampened in black flying foxes in comparison to humans, due to the replacement of a highly conserved serine residue (S358) with a histidine (S358H) in the stimulator of IFN genes (STING) protein [59], which is thought to ultimately limit IFN-β production [60]. Similarly, Jamaican fruit bats and Egyptian fruit bats exhibit the S358H substitution, whereas the large flying fox harbors a tyrosine (S358Y) and the little brown bat an asparagine (S358N) at this position [60]. However, whether the IFN signaling is likewise dampened in these species needs to be addressed. Recent in vitro studies have also revealed that the black flying fox and David’s myotis bat (*Myotis davidii*) have a dampened NLRP3-inflammasome activation, accompanied by either a reduction in caspase-1 or IL-1β activation, while NF-κB signaling and its regulated genes (*Il1β*, *Il6,* and *Tnfα*) appeared to be unaffected [61,62]. Accordingly, an increase in cytokine transcripts (*Il6*, *Il8*, *Il1a*, *Il1b*, and *Ifng*) were observed in Jamaican fruit bats upon infection [21].

Aside from the innate immune response, intriguing features of Jamaican fruit bat adaptive immunity could be identified. As in all vertebrates, viral infections were found to trigger a humoral immunity, although titers varied depending on the virus, as described above. As described for other bat species [63,64,65,66,67], four immunoglobulin isotypes (IgM, IgE, IgA, and IgG) were expressed in the Jamaican fruit bat (Table 1) [21]. Interestingly, IgD appears to be specific for insectivorous bat species, and is thus not encoded in Jamaican fruit bats, as well as other frugivorous species genomes, such as the New World Seba’s short-tailed bat [58,65], the South Asian greater short-nosed fruit bat *(Cynopterus sphinx)* [65], the black flying fox, and the large flying fox [45,63]. Only one subclass of IgG was identified in both Jamaican fruit bats and Seba’s short-tailed bats [21], which is in contrast to the four distinct IgG subclasses known for humans, and the multiple subclasses of IgG found in mice, as well as little brown bats, big brown bats *(Eptiscus fuscus),* and the greater short-nosed fruit bat [65] (Table 1). Intriguingly, upon TCRV infection of Jamaican fruit bats, it was shown that even though all classes of antibodies were upregulated, the transcription of activation-induced cytidine deaminase (AID), a protein involved in class-switch recombination and somatic hypermutation, was not. Nevertheless, the Jamaican fruit bat transcriptome data indicated a high diversity in the heavy-chain variable (VH) regions of antibodies [21,58]. This is in line with previous studies in little brown bats and black flying foxes, suggesting that combinatorial diversity early in antibody production could potentially compensate for the less important role of somatic hypermutation and affinity maturation [58,63,68]. Interestingly, little brown bats were similarly shown to have a diverse VH repertoire and a lack of somatic hypermutation [68]. The black flying fox, despite harboring a diverse VH repertoire, was shown to use somatic hypermutation, though to a lower degree than known for humans [63,69]. In addition to variations in antibody production, the constellation of adaptive immune cells involved in the antiviral response appears to differ to that of humans and across bat species. Although activation markers for T and B lymphocytes were upregulated upon infection of Jamaican fruit bats, the same was not true for natural killer (NK) cells, indicating that they may not be as important as in humans [21]. In line with this, the sequencing of the Egyptian fruit bat genome revealed that many of the receptor complexes involved in NK cell regulation and inhibition, including the killer cell immunoglobulin-like receptors (KIRs) [70,71], were not present [49]. Furthermore, those that were conserved, including multiple killer lectin-like receptors (KLRs) that were also identified in the black flying fox [45], tended to contain inhibitory motifs, which may be indicative of a reduced role for NK cells in these species as well [46,49]. Previous studies in the black flying fox using cell-specific antibodies have shown that the makeup of lymphocyte cell populations can vary widely between species [72,73]. In humans, B cells and T cells constitute 3–15% and 40–60%, respectively, of peripheral blood mononuclear cells (PBMCs). In black flying foxes, the percentage is higher for both cell types (B cells: 29–33%, T cells; 62–64%) [72]. Follow-up studies in the cave nectar bat *(Eonycteris spelaea)* showed comparable B and T cell percentages in the spleen, bone marrow, and blood. However, cave nectar bats completely lacked NK cells in both the spleen and bone marrow, despite the fact that this cell population was prominent in the spleen and bone marrow of the black flying fox [73]. Additional experiments are required to determine which of these cells are involved in the antiviral adaptive immune response of Jamaican fruit bats, which has so far been limited by the lack of cell-specific antibodies [17,21]. Especially for the study of H18N11, which replicates in immune tissue, markers for these different cell types will be particularly useful.

Therefore, to fully understand their response to viral infection, future experiments need to address the missing information about Jamaican fruit bat immunity. First, it should be determined whether the innate immune response in these bats is dampened upon infection. This includes characterizing baseline IFN levels, as well as components of the STING and inflammasome activation pathways, both before and upon infection. Additionally, organ-specific and cell-specific differences in the antiviral response need to be taken into account. Future transcriptome studies should therefore focus not only on gene expression in infected organs, but in individual infected cells as well. Conversely, the immunomodulatory functions of viral proteins, such as the bat IAV IFN antagonist NS1 [74,75,76,77], should be considered when interpreting the Jamaican fruit bat immune response to infection.

Taken together, the observations made for different bat species indicate that many generalizations about bat immunity are unlikely to hold true and suggest a more varied immune response to viral infection. This is not surprising, considering there are more than 1400 species of bats in the order Chiroptera that emerged at least 64 million years ago [78]. Given this great immunological variance, it is becoming clear that different bat species and their individual immune responses co-evolved with the respective viriome they harbor. To accurately study the bat immune system, an authentic virus-host setting should be utilized. With regard to Jamaican fruit bats, further research using the appropriate model viruses will fill in the remaining gaps in the current knowledge of its immunity and, moreover, explore viral mechanisms, particularly immunomodulatory functions, which might allow viruses to enter host cells, replicate, and be transmitted among this bat species without causing significant damage.

## Figures and Tables

**Table 1:** Summary of the identified immunoglobulin isotypes and the number of subclasses in the indicated species.

Species	Immunoglobulin Isotypes				
	IgM	IgE	IgA	IgG	IgD
*H. sapiens*	1	1	1	4	1
*M. musculus*	1	1	1	5	1
*R. aegyptiacus*	1	2	1	4	-
*P. alecto*	≥1	1	1	≥1	-
*P. vampyrus*	1	1	≥1	≥1	-
*M. lucifugus*	1	1	1	5	1
*E. fuscus*	1	1	1	2	1
*C. sphinx*	1	1	1	3	-
*C. perspicillata*	1	1	1	1	-
*A. jamaicensis*	1	1	1	1	-

## Supplementary Materials

The following supporting information can be downloaded at: https://www.mdpi.com/article/10.3390/v14020238/s1, Figure S1: *A. jamaicensis* interferon lambda-like transcripts.
