# Supplementary material for: Different but Not Unique: Deciphering the Immunity of the Jamaican Fruit Bat by Studying Its Viriome
Source: Viruses. 2022 Jan 25;14(2):238. doi: 10.3390/v14020238 (PMC8879847; doi:10.3390/v14020238)
Supplement: Supplementary file 1 [file viruses-14-00238-s001.zip › viruses-1523317-supplementary.pdf]

Figure S1 – *A. jamaicensis* interferon lambda-like transcripts

Nucleotide sequences and the respective amino acid sequences of the translated open reading frames (ORFs) for IFN lambda-like transcripts (**A**) and the components of type III IFN receptor, IFNLR1 (**B**) and IL10RB (**C**), identified in Jamaican fruit bat transcriptomic data (BioProject PRJNA305413) are presented. The amino acid sequence of the transcriptome sequences were aligned to the genomic data (BioProject PRJNA673233) retrieved from the NCBI genome database.

A.

Transcript: IFN lambda-3-like (comp418173_c0_seq3, missing first 27 residues)

ccaggagccacaggctgtaccacacagtccaggtgtgtggtgacggccccatcgcggtcagtgtgagtgctctgtgacgtctgcacaacgacaggttcccctaaggacgcattcctcaaaaggactcctggtggt
tacatggctggggactgtaactgagcctgctgacctcggtgctctccaaatccctcagaaatgggacccgctcagtggggagagccaacagtcagagactcacagaacatccagggaggcgttcaggggaaac
aagactggtccatggtccatggggtcagcaccaaagtggttccagaattcaggcctctcccaaaccagccctggaaactgaatccccccacacactccccacagcacaaacagcatcttttcctcattgtttatttgca
caatttgacaaaaagtaaaaaaataaacacttaattggttgtgagtttgaaaattcacatggataaatacataaataactgacaataacttaaaaagtgaatgaggcataaataaaatctcctgattgggtgtgagtt
tgaaaatgcccacatacaaatatataaataactgacaataacttaagaaaaatgagtgaggcataaataacatctaagatgctggggcaggtggcgggtgggcctcagggtcagacacacaggtctccactggc
gacacagttcaggtcatgggtgaggaggcggaacaggttgaatgtcacagaggcttcaaggcaggcaggggactcctttgaagccacctggaggcggcgcagccagcggtggaggtagccccggggtctg
gggcctgctgtggcctgagctgggacacaggcctgaagcttggtgtggatgtagcgcagcatgtgaaggggctgctccaggatgtcccccagggtggagttagccatggtccccaggaccttcagtgtcaggg
ccagctcagcttccaaggccactgggcgctcccatacctgcagctgcctcaggtcccgggtcctgggcagtgggtgggagctgcagctccagttcttcagcaggagtgactcctccaaggcgtccttggccctcct
gaaggccttcatgtcttgtgggggcagagacttgaactgggccatggggcagtcccttacacccgggagggcactgaggggcgtggggacaggaactgctcccgtcctgctcatcactgtgatcatcagcatca
gcaccagcatgcaacccacaggcaagtccagctggatcctcatctctgtctctggtgtctgtcagcagagggggacccaagtgtccactgagaggctgtcaccacggtccctcactcagccttgtttggtgtctgtg
acctcagtctcctctgctgggtctgagtctgctgctctgtctgcgtctctgcacctccagctctgctcagatggaacagctgcctgagccccatggtatagcttttagcctggagagatgggcaatcccagctgatgta
ggaaaagtgaaaacacactggccactaggggaggccccaggctg

ORF:

QTPETEMRIQLDLPVGCMLVLMLMITVMSRTGAVPVPTPLSALPGVRDCPMAQFKSLPPQDMKAFRRAKDALEESLLLKNWSCSSHPLPRTR
DLRQLQVWERPVALEAELALTLKVLGTMANSTLGDILEQPLHMLRYIHTKLQACVPAQATAGPRPRGYLHRWLRRLQVASKESPACLEASV
TFNLFRLLTHDLNCVASGDLCV

Alignment to: interferon lambda-3-like [Artibeus jamaicensis] Sequence ID: XP_036985040.1

```
GSE75771_PRJNA305413_(transcriptome)    DLPVGCMLVL MLMITVMSRT GAVPVPTPLS ALPGVRDCPM AQFKSLPPQD MKAFRRAKDA LEESLLLKNW SCSSHPLPRT
-                                       DLPVGCMLVL MLMT-VMSRT GAVPVPTPLS ALPGR--CPM AQFKSLPPQD MKAFRRAKDA LEESLLLKNW SCSSP--PRT
GCA_014825515.1_PRJNA673233_(genome)    DLPVGCMLVL MLMTTVMSRT GAVPVPTPLS ALPGARGCPM AQFKSLPPQD MKAFRRAKDA LEESLLLKNW SCSSYPFPRT

GSE75771_PRJNA305413_(transcriptome)    RDLRQLQVWE RPVALEAELA LTLKVLGTMA NSTLGDILEQ PLHMLRYIHT KLQACVPAQA TAGPRPRGYL HRWLRRLQVA
-                                       RDLRQLQVWE RPVALEAELA LTLKVLGTMA NSTLGDILEQ PLH-LRYIHT KLQACVPAQA TAGPRPRGYL R-WLR---LA
GCA_014825515.1_PRJNA673233_(genome)    RDLRQLQVWE RPVALEAELA LTLKVLGTMA NSTLGDILEQ PLHTLRYIHT KLQACVPAQA TAGPRPRGYL RRWLRHLEEA

GSE75771_PRJNA305413_(transcriptome)    SK-ESPACLE ASVTFNLFRL LTHDLNCVAS GDLCV
-                                       SK-ESPACLE ASVTFNLFRL L--DLNCVAS GDLCV
GCA_014825515.1_PRJNA673233_(genome)    SKRESPACLE ASVTFNLFRL LARDLNCVAS GDLCV
```

B.

Transcript 1: IFNLR1 (comp399499_c0_seq8 frame 6, missing last 23 residues)

tcctcctcctcctcctcctccccgtcctcggtgctgctgtcccagccaaaggtcagtgtctgaaaagaaactagaggctccccggggaccagtttcagtcttggtgatgaggagccccagtcagcccaggaggaga
ggtcatctctctggagctctttcagggaacccaagtccttggagaatttggggagtgggagagggtcagacctgtcctcacccagccctcggtccagacccttcttgactaagtagccagaggatccagccccatct
gagagggaggagccgacggtgctggcccagcttctgtctgaagaatccgaagaaggacagccttcgacctggacccctccctcgggttgctccggggtctggtgctcctgctccaggaaaggagggagtgca
acatagggctggaagctgacattgtcgtctgtgtcctcctcgctgtcctcttccttagcaccgtccttctctgatcctgcctggacggtggctggggccctggccctggaggtcagtttgatccttctggtcagttcctttt
ggggacagagggtcaagccatccaggggctcggggccacagggctgaaaggttgccacggggtgtctgtgtccagaaaagtccagagcctgtggcatctctgcccgctgaaaccaggggttccccgcgtagc
tcttccagatcacccagctcttccagatcacacatgccacaaggagagccaacagcagtggcagcagcaccagggacgcccagctgactcctggggtctccagggagaagcaggtgggcttggagaactcgct
gtatttaggatcgcccaaggtgtagattgttctggcgctgacgcagtggttcccactggtacctggctggagggggatctggacttcctggccgtggagggtggctggaagtcgggtcttgttcccagtggcctcc
ttccagaaatccacctcgtacttcagattcgatgagggcatgcagtggggcagctggtacgtggcggtgaccttcaggatctcctctgtcagagtgactaccaggacaggcggggccggctccacttcaaaaagg
tactccaggtactcggactccacccaggtggacctggcgctgggggaaaccgcccgcacacgccccttgaacttgttgaataggtcctgcttctccaggcacatcagagagcatgtcagctctttggttcctgcaca
ctttcccactttccgccaccgtctgggggttgttgcggagctttgataagccacgagataggtcacgtcctgcgggttgccaggccctgggagccatgtcaggtacacactgaagttccgggacatcagtgtcacgt
tctggggaggggccagatggggcctccctccggcggactgcagtagagagacgagcaggagggcccagcgtccggccgggctcatgtcagtccttccccggcccgccgggcgggcgggat

ORF:

MSPAGRWALLLVSLLQSAGGRPHLAPPQNVTLMSRNFSVYLTWLPGPGNPQDVTYLVAYQSSATTPRRWRKVGKCAGTKELTCSLMCLEK
QDLFNKFKGRVRAVSPSARSTWVESEYLEYLFEVEPAPPVLVVTLTEEILKVTATYQLPHCMPSSNLKYEVDFWKEATGNKTRLPATLHGQEV
QIPLQPGTSGNHCVSARTIYTLGDPKYSEFSKPTCFSLETPGVSWASLVLLPLLLALLVACVIWKSWVIWKSYAGNPWFQRAEMPQALDFSGH
RHPVATFQPCGPEPLDGLTLCPQKELTRRIKLTSRARAPATVQAGSEKDGAKEEDSEEDTDDNVSFQPYVALPPFLEQEHQTPEQPEGGVQVE
GCPSSDSSDRSWASTVGSSLSDGAGSSGYLVKKGLDRGLGEDRSDPLPLPKFSKDLGSLKELQRDDLSSWADWGSSSPRLKLVPGEPLVSFQTL
TFGWDSSTEDGEEEEEE

Alignment to: interferon lambda receptor 1 [Artibeus jamaicensis] Sequence ID: XP_036991817.1

```
GSE75771_PRJNA305413_(transcriptome)    MSPAGRWALL LVSLLQSAGG RPHLAPPQNV TLMSRNFSVY LTWLPGPGNP QDVTYLVAYQ SSATTPRRWR KVGKCAGTKE
-                                       MSPAGRWALL LVSLLQSAGG RPHLAPPQNV TLMSRNFSVY LTWLPGPGNP QDVTY-VAYQ SSA-TPR-WR KVGKCAGTKE
GCA_014825515.1_PRJNA673233_(genome)    MSPAGRWALL LVSLLQSAGG RPHLAPPQNV TLMSRNFSVY LTWLPGPGNP QDVTYRVAYQ SSA-TPQRWR KVGKCAGTKE

GSE75771_PRJNA305413_(transcriptome)    LTCSLMCLEK QDLFNKFKGR VRAVSPSARS TWVESEYLEY LFEVEPAPPV LVVTLTEEIL KVTATYQLPH CMPSSNLKYE
-                                       LTCSLMCLEK QDLFNKFKGR VRAVSS--RS TWVESEYLEY LFEVEPAPPV LVVTLTEEIL KV-ATYQLPH CMPSSNLKYE
GCA_014825515.1_PRJNA673233_(genome)    LTCSLMCLEK QDLFNKFKGR VRAVSASTRS TWVESEYLEY LFEVEPAPPV LVVTLTEEIL KVNATYQLPH CMPSSNLKYE

GSE75771_PRJNA305413_(transcriptome)    VDFWKEATGN KTRLPATLHG QEVQIPLQPG TSGNHCVSAR TIYTLGDPKY SEFSKPTCFS LETPGVSWAS LVLLPLLLAL
-                                       VDFWKEATGN KTRLPATLHG QEVQIPLQPG TSGNHCVSAR TIYTLGDPKY SEFSKPTCFS LETPGVSWAS LVLLPLLLAL
GCA_014825515.1_PRJNA673233_(genome)    VDFWKEATGN KTRLPATLHG QEVQIPLQPG TSGNHCVSAR TIYTLGDPKY SEFSKPTCFS LETPGVSWAS LVLLPLLLAL

GSE75771_PRJNA305413_(transcriptome)    LVACVIWKSW VIWKSYAGNP WFQRAEMPQA LDFSGHRHPV ATFQPCGPEP LDGLTLCPQK ELTRRIKLTS RARAPATVQA
-                                       LVACVIWKSW VIWKSY-GNP WFQRAEMPQA LDFSGHRHPV ATFQPCGPEP LDGLTLCPQK ELTRRIKLTS RARAPATVQA
GCA_014825515.1_PRJNA673233_(genome)    LVACVIWKSW VIWKSYVGNP WFQRAEMPQA LDFSGHRHPV ATFQPCGPEP LDGLTLCPQK ELTRRIKLTS RARAPATVQA

GSE75771_PRJNA305413_(transcriptome)    GSEKDGAKEE DSEEDTDDNV SFQPYVALPP FLEQEHQTPE QPEGGVQVEG CPSSDSSDRS WASTVGSSLS DGAGSSGYLV
-                                       GSEKDGAKEE DSEEDTDDNV SFQPYVALPP FLEQEHQTPE QPE-GVQVEG CPSSDSSDRS WASTVGSSLS DGAGSSGYLV
GCA_014825515.1_PRJNA673233_(genome)    GSEKDGAKEE DSEEDTDDNV SFQPYVALPP FLEQEHQTPE QPEEGVQVEG CPSSDSSDRS WASTVGSSLS DGAGSSGYLV

GSE75771_PRJNA305413_(transcriptome)    KKGLDRGLGE DRSDPLPLPK FSKDLGSLKE LQRDDLSSWA DWGSSSPRLK LVPGEPLVSF QTLTFGWDSS --TEDGEEEE
-                                       KKGLDRGLGE DRSD-LPLPK FSKDLGSLKE LQRDDLSSWA DWGSSSPRLK LVPGEPLVSF QTLTFGWDS- --TEDGEEEE
GCA_014825515.1_PRJNA673233_(genome)    KKGLDRGLGE DRSDLLPLPK FSKDLGSLKE LQRDDLSSWA DWGSSSPRLK LVPGEPLVSF QTLTFGWDSG TGTEDGEEEE

GSE75771_PRJNA305413_(transcriptome)    EE
-                                       EE
GCA_014825515.1_PRJNA673233_(genome)    EE
```

Transcript 2: IFNLR1, 3′ end (comp361833_c0_seq2, frame 6)

attcaattcttccttttctctaaaaatagagctctttatttaaaagtacagagttctaaaatgagcattaagaaattatttaaatatgatataaacatataaagttagaaataaatacaaaaatctaagaataatatataat
ctcagtacagctgacccttgaatgacatgggtttgaactgtaatgggtccatttagacgcagatttttctcaataaatacacaatcagccctctgtatctctgggttttgcattgaacgaaaacagtatttttgatccttgg
ttgggaatttgcggatgcggtggacagacttgagcatgcgtggatttgggtatctgcggatgcagggctggtggggggcattcctggaaccaatcccctgcagatactgaggtatgaccgaagtttccgggacc
caaaagttatacgtgaatgctcgactgtgtggtggggtcgtgcccctaatcccttcaagagtcaactgtgctctgaaaaattataagttgctgcaaccaaaggcatcaggtattcgtgacccgctgtccctcatcacg
gccctggggctggttgtgcccaccaagggcaagttattatgatgtttcccacagtttttcatgatacaatcactgtaaaggagctggaatttaaaattactaaaaagctcagcttatctgtgaaattaaatgttgaaaa
aaagctttaaaagaaccccaacgattagtaataataaagataatgtgattcctttaccagagtgctaatggctttcagtttttgaaattaccttcaggaagcagctcatctgggttccagagagaacttcagcctcagtc
ggcattttcccaggaggaggccccgagccacagccttgcccagcaaggagacttcagtgcgttcttcctcccctgcgctcagcgatgtggacaagccttcacccctgtgcgagcacacacacgtgtgcatgcgtgt
gcatatgcctgtgagccccagcagcgaaggcctcctgccaactttgtgcccccaggaagtccttgctgtgacctcagctgtttgctgccatggccaagggaacgggggacggaccagctgggcacagaccgcaa
agctcaccctcctggagaaacatggtgggtcagctgctcagctgcttcagacagaagtctggaccctgagggtgaagactctgccgtgcttgtccgtactcgggtgggggtaggttaatgaagccagcacccatc
actaccgctaatggcctgtttctggaagacactgacagggaagatgggactttttttataaccacagtggccagccagagggtcaaagcattgtccgaagcagggttacacccaggggtcccattcttgtctggct
gctgtcccatccccagcaaactggaacccctggaagacgcaccgtttgtttttcactgggcaaagggagagggaccaagagcccccctgggctgggtttcctgaccaggaggtggagacggcgaaggtggaa
gacaggaggtggacgagaagtgagaccggctgaggggtgagtgctcccaggccctctgagttgaaggccatggcgggagacttccggcagtctccccagagcccgggcagatggagaaccccccagcccc
caggaagagagcaccagtgatgggcaggctcccccacactccaggtggaagtgcaggcgaggaaaccctagggcagagaaggaagctgcttacccaggtcacatgactgggcaatggcagagcagggat
cggcccaggttggcctgatagcaaacctgtgcagttccatgacagagacacagatgcggctgatggctgcccttcccttcccctcgaacactgtctgtcaacgaggttcttcctcactgtggcccgccctaggggac
tcacgaatcaggagcccaagacttggtggagagggcgctggagaaaggcaggggctccgcactgccgtgcctccgaacatcctgctctcaacccagatggcccctggtggtgtcagccctggatgtctactgtg
acccggccacgcctgtgtccagggatgatttttccacctgacctttcaccccaggcacagtggggtgagcccagcccatcctcttctccagtctttctggagctccacccatagctggtgggtccaagggcgaggtg
gctccttaccgctcaacgggacagatggacaaacggcctctagctggggtttgggaagcgctatgagaacactgtgcctgctacccccgggatgatgctctcaggctttcaagtctcagcggcatcttgggccctt
gggaagctcagtagcaccggatttggcaggaagctgggggacagctcacctggccaggtaatgccccagtgtcctactcctttgaaggctgtcagtcccccagctgccagcactggtgtcctcaagttctgattcg
ctcccaccctcttcctcctcctcctcct

ORF:

EEEEEEGGSESELEDTSAGSWGTDSLQRSRTLGHYLAR

Alignment to: interferon lambda receptor 1 [Artibeus jamaicensis] Sequence ID: XP_036991817.1

```
GSE75771_PRJNA305413_(transcriptome)    DTSAGSWGTD SLQRSRTLGH YLAR
-                                       DTSAGSWGTD SLQRSRTLGH YLAR
GCA_014825515.1_PRJNA673233_(genome)    DTSAGSWGTD SLQRSRTLGH YLAR
```

C.

Transcript: Interleukin-10 receptor subunit beta (complete CDS, frame 5)

gggcccttctgttctcccaccttccagcaccgggagctcccaggtgcggccgggccccgccccaccctggctcccagctaacagacagcggtggggggagggactgtgggactcccactgggcaaacgcacag
catcaacagctctcaagagccttgcccggcaccggacagcctgcaaatgcaaggtcaggaacacactcagggtggagaataaaactttaatggaggccggcgctgtgctggacgtgggcggggaggaaaac
aaagccccgggaaccgaagcccgcactctgcacagcgagtttctggggtcgcgggccggccgtcagtgtgacgtcatggcacctgttttcactcctcaccgtaagtatgttcggttctcgcaattaaaactgttcctt
ccttcaggcagtgagcctgacaggctgtaacgacagccctggtgggcgaggcctttgccgggcagctggttcccaggtggaggcctctggccaagcgagcctttcaagaccagtgctggctgttcagacgcgg
cccggggtcccgggttccagaccgggcctggctcggggcgtctgggccccccaccaccgctgctcctattgccctccgaggtggctgtgagatgcaggacctgccccctcctcagggactaggtggcaggcgg
gtcccacgccgaaggcggcccgaggccacagtcgtccccggcatcccgcccgctgcctttggggagctcggcgacgacgctcagtctgtcgaagacttcgctctcatcggggcacgggaaggagacgaacag
cagcctgttgcgatgcggctggttcagaaactccttcaggtgctgcgggagcccgtcccccgggagaagatgcgcctggtcctcttgtagacgcaccacagcagggcggagcaccccagcagcagcaggca
ggtggcgcacaccgaggcccccagcacggcggccaccacgacccaggagggggctgcttcggcactggaggtctgctcgcagacaggctcgctccactccccggccttgttctgatcgagaagaaacccctg
gacgcgaacacagtatgtcgtccagggctccaggtttcccacgacctcgaagtcacgctgacaagcaactgtgtgcttctcgtcagagccattcttccaatactgcacattgtagacccacgagtcgtagaagttcct
catggtccaggtttcatgctccttctcaattctgggggctgagaaacgtatgtggaaagaattggcaactgcttctacgtgtattcctggaggtccaatgtcggtgtcgtccaccggacagaagatgagggtcaccc
actgggagtgctcgtctgcctgctccgccctgactctcaaggtgtggttgccgtacttggacagcccggagaagtcacaccgagtcagggcagtgccggtgcacacgtcctggaattccttgtaactgaagtactg
agccgtgaaagtcagattccctttgggaaaagcaggaggctcccattctagaatgttcttgaagttaactgaattcattctgacgttttcaggaggagggaccattcctaatgctgacaccaggaggcagctgccta
gccagctccggaggctgcgcgccatgcccgcgcagggagccccgagggcgcgggggcagtgggcataggtccccgagtccccttggtgccgcgacactgtggccggcccagcgccata

ORF:

MALGRPQCRGTKGTRGPMPTAPRALGAPCAGMARSLRSWLGSCLLVSALGMVPPPENVRMNSVNFKNILEWEPPAFPKGNLTFTAQYFSY
KEFQDVCTGTALTRCDFSGLSKYGNHTLRVRAEQADEHSQWVTLIFCPVDDTDIGPPGIHVEAVANSFHIRFSAPRIEKEHETWTMRNFYDS
WVYNVQYWKNGSDEKHTVACQRDFEVVGNLEPWTTYCVRVQGFLLDQNKAGEWSEPVCEQTSSAEAAPSWVVVAAVLGASVCATCLLLL
GCSALLWCVYKRTRRIFSRGDGLPQHLKEFLNQPHRNRLLFVSFPCPDESEVFDRLSVVAELPKGSGRDAGDDCGLGPPSAWDPPAT

Alignment to: interleukin-10 receptor subunit beta [Artibeus jamaicensis] Sequence ID: XP_036983084.1

```
GSE75771_PRJNA305413_(transcriptome)    MALGRPQCRG TKGTRGPMPT APRALGAPCA GMARSLRSWL GSCLLVSALG MVPPPENVRM NSVNFKNILE WEPPAFPKGN
-                                       MALGRPQCRG TKGTRGPMPT APRALGAPCA GMARSLRSWL GSCLLVSALG MVPPPENVRM NSVNFKNILE WEPPAFPKGN
GCA_014825515.1_PRJNA673233_(genome)    MALGRPQCRG TKGTRGPMPT APRALGAPCA GMARSLRSWL GSCLLVSALG MVPPPENVRM NSVNFKNILE WEPPAFPKGN

GSE75771_PRJNA305413_(transcriptome)    LTFTAQYFSY KEFQDVCTGT ALTRCDFSGL SKYGNHTLRV RAEQADEHSQ WVTLIFCPVD DTDIGPPGIH VEAVANSFHI
-                                       LTFTAQYFSY KEFQDVCT-T ALTRCDFSGL SKYGNHTLRV RAEQADEHSQ WVTLIFCPVD DTDIGPPGIH VEAVANSFHI
GCA_014825515.1_PRJNA673233_(genome)    LTFTAQYFSY KEFQDVCTST ALTRCDFSGL SKYGNHTLRV RAEQADEHSQ WVTLIFCPVD DTDIGPPGIH VEAVANSFHI

GSE75771_PRJNA305413_(transcriptome)    RFSAPRIEKE HETWTMRNFY DSWVYNVQYW KNGSDEKHTV ACQRDFEVVG NLEPWTTYCV RVQGFLLDQN KAGEWSEPVC
-                                       RFSAPRIEKE HETWTMRNFY DSWVYNVQYW KNGSDEKHTV ACQRDFEVVG NLEPWTTYCV RVQGFLLDQN KAGEWSEPVC
GCA_014825515.1_PRJNA673233_(genome)    RFSAPRIEKE HETWTMRNFY DSWVYNVQYW KNGSDEKHTV ACQRDFEVVG NLEPWTTYCV RVQGFLLDQN KAGEWSEPVC

GSE75771_PRJNA305413_(transcriptome)    EQTSSAEAAP SWVVVAAVLG ASVCATCLLL LGCSALLWCV YKRTRRIFSR GDGLPQHLKE FLNQPHRNRL LFVSFPCPDE
-                                       EQTSSAEAAP SWVVVAAVLG ASVCATCLLL LGCSALLWCV YKRTRRIFSR GDGLPQHLKE FLNQPHRNRL LFVSFPC-DE
GCA_014825515.1_PRJNA673233_(genome)    EQTSSAEAAP SWVVVAAVLG ASVCATCLLL LGCSALLWCV YKRTRRIFSR GDGLPQHLKE FLNQPHRNRL LFVSFPCSDE

GSE75771_PRJNA305413_(transcriptome)    SEVFDRLSVV AELPKGSGRD AGDDCGLGPP SAWDPPAT
-                                       SEVFDRLSVV AELPKGSG-D AG-DCGLGPP -SWDPPAT
GCA_014825515.1_PRJNA673233_(genome)    SEVFDRLSVV AELPKGSGWD AGDVCGLGPP SGWDPPAT
```
